# Consumption of Dairy Foods and Cardiovascular Disease: A Systematic Review

**DOI:** 10.3390/nu14040831

**Published:** 2022-02-16

**Authors:** Annalisa Giosuè, Ilaria Calabrese, Marilena Vitale, Gabriele Riccardi, Olga Vaccaro

**Affiliations:** 1 Department of Clinical Medicine and Surgery, “Federico II” University of Naples, 80131 Naples, Italy; annalisa.giosue@gmail.com (A.G.); ilariacalabrese@live.it (I.C.); marilena.vitale@unina.it (M.V.); riccardi@unina.it (G.R.); 2 Department of Pharmacy, “Federico II” University of Naples, 80131 Naples, Italy

**Keywords:** dairy foods, cheese, yogurt, cardiovascular disease, cardiovascular risk factors

## Abstract

Limited consumption of dairy foods and use of low-fat products is recommended for cardiovascular (CV) prevention; however, other features besides fat content modulate their metabolic effects. We analyze updated evidence on the relationship of different dairy products (low/full-fat dairy, milk, cheese, yogurt) with CVD by reviewing meta-analyses of cohort studies and individual prospective cohort studies with CV hard endpoints (CVD/CHD incidence/mortality), together with meta-analyses of randomized controlled trials exploring the effect of dairy on major CV risk factors. The analyses provide evidence that moderate dairy consumption (up to 200 g/day, globally) has no detrimental effects on CV health and that their effect depends more on the food type (cheese, yogurt, milk) than on the fat content. These data expand current knowledge and may inform revision of current guidelines for CVD prevention.

## 1. Introduction

Cardiovascular diseases (CVDs) constitute the leading cause of global mortality and are a major contributor to reduced quality of life, and thus are a key challenge to health care systems [1,2]. Dietary patterns and particularly fat intake have long been implicated in the modulation of the cardiovascular (CV) risk [3]. A high intake of saturated fatty acids (SFAs) and trans-fatty acids (TFAs) has been linked to an enhanced risk of CVD, and this effect is thought to be mediated predominantly by increased plasma LDL cholesterol levels and their proatherogenic effect [4,5,6]. The most recent guidelines of the European Society of Cardiology (ESC) and American Heart Association (AHA) recommend <10% of total energy intake from SFAs, yet most developed countries currently exceed this recommendation [7,8].

Recent research has emphasized the importance of focusing on whole foods rather than single nutrients when exploring the relationship of nutrition with disease risk [9].

This is justified by the observation that the physical form of the food, the specific combination of macro/micronutrients and non-nutrient bioactive compounds within each food can play an important role in modulating the metabolic effect of foods and their impact on health beyond their nutrient’s composition. Furthermore, detailed information on the associations between each food item and health outcomes can also facilitate the translation of the evidence derived from nutritional research into clinical recommendations, thus improving adherence [10,11,12,13].

Among the foods that represent a major source of dietary SFAs, dairy foods deserve consideration since they are largely consumed worldwide [14] and contribute a relevant proportion to the global SFAs intake (i.e., one fifth in the USA, 17–41% in Europe) [15]. A substantial reduction in the intake of dairy and a preferential consumption of low-fat products has been advocated as a strategy for CVD prevention, although evidence in this regard is scant and inconsistent. Dairy foods are a highly heterogeneous food group comprising foods with different biochemical composition, nutritional characteristics (i.e., fat, micronutrients, and salt content), preparation techniques (i.e., fermentation, pasteurization, processed by enzymatic procedures) which may all impact on their nutritional properties and metabolic effects. Partly due to this heterogeneity, the relationship of dairy consumption with cardiovascular diseases remains controversial. The large geographical variation of patterns of dairy food consumption, deeply rooted in sociocultural behaviors and, therefore, influenced by the background diet, further adds to the complexity of the relationship of dairy consumption with health outcomes.

It is therefore appropriate to evaluate the updated evidence on the relationship between dairy food consumption and CVD taking into account the available information on the specific food items included in the dairy food group; in particular, it is relevant to evaluate the impact of dairy foods on established and emerging cardiovascular risk factors in order to substantiate possible mechanisms trough which dairy foods may impact on CV health and differentiate population groups that might be more prone to their possible untoward effects.

Against this background, to shed light on the complex relationship between dairy intake and CV health, we have reviewed the literature on the relationship between the consumption of total dairy foods, or single dairy food items, and CV hard endpoints (i.e., CVD and coronary heart disease (CHD) incidence/mortality) as well as all-cause mortality, focusing on meta-analyses of prospective studies, since they represent a comprehensive and weighted synthesis of the available evidence. Furthermore, to substantiate possible cause–effect relationships and investigate biologically plausible mechanisms through which dairy may have an impact on CVD risk, we have reviewed the available meta-analyses of randomized controlled trials (RCTs) exploring the effect of the various dairy products (i.e., low/full-fat dairy, milk, butter, cheese, yogurt) on major cardiovascular risk factors. By fulfilling the study aims we expect to contribute to a better understanding of the complexity of the relationship between nutrition and CV health.

## 2. Materials and Methods

### 2.1. Meta-Analyses of Prospective Cohort Studies on Cardiovascular Hard Endpoints and All-Cause Mortality: Literature Search Strategy and Data Extraction

We performed a systematic literature search in PubMed, Embase, Scopus, and Cochrane Library databases for meta-analyses of prospective cohort studies examining the association between dairy food consumption and all-cause mortality as well as CVD/CHD incidence and mortality according to PRISMA (Preferred Reporting Items for Systematic reviews and Meta-Analyses) guidelines [16], up to 30 April 2021. We used various combinations of the following keywords: “dairy”, “dairy products”, “total dairy”, “full fat dairy”, “low fat dairy”, “milk”, “fermented dairy”, “cheese”, “yogurt”, “all-cause mortality”, “coronary heart disease incidence”, “coronary heart disease mortality”, “cardiovascular disease incidence”, “cardiovascular disease mortality”, “coronary artery disease”, “acute myocardial infarction”. The full details on the search strategy are presented in the Online Supplementary Materials (Supplemental Methods S1). We also performed an additional manual search through the reference lists of original publications to identify further pertinent studies. The search was limited to meta-analyses of human studies and was restricted to papers written in English.

Studies were considered for inclusion in the present systematic review if they met the following criteria: (1) the authors reported data from an original, peer-reviewed study (not reviews, conferences, and letters); (2) the study had a prospective design; (3) the authors reported RRs, HRs, or ORs with 95% CIs for dairy food consumption; (4) the investigators reported ≥1 of the outcomes of CVD risk, including incidence of total CVD and CHD, or CVD and CHD mortality, or all-cause mortality. We included only prospective cohorts to minimize recall and selection bias. Exclusion criteria were meta-analyses of cross-sectional and/or case-control studies and meta-analyses of prospective cohort studies conducted on populations with special dietary habits (i.e., vegetarians, vegans) or with physiological or pathological conditions requiring specific dietary treatment (i.e., pregnancy or breastfeeding, childhood, diabetes, dyslipidemia, hypertension, etc.).

Two investigators (IC and AG) independently conducted a 2-stage selection process to identify eligible studies: an initial screening of titles and abstracts, followed by an evaluation of all potentially relevant full-length articles. Any discrepancy was resolved by discussions with another investigator (MV). Studies were excluded if they failed to meet the criteria detailed above.

The reason to focus on prospective studies was that this study design allows a more successful control for confounding factors and represents the best source of evidence when RCTs are not available. Furthermore, the utilization of meta-analyses allows a comprehensive and weighted summary of the available evidence. Results of prospective studies not included in the reviewed meta-analyses were also evaluated and reported when relevant for the aims of this review.

The association of dairy consumption with the outcomes was generally estimated by comparing the highest with the lowest level of consumption since a minority of studies report dose–response analyses; where available, these data were collected in order to identify the serving size associated with the best outcomes or representing the threshold of intake above or below which the relationship curve departs from linearity.

### 2.2. Meta-Analyses of RCTs on the Effect of Dairy Products on Major CV Risk Factor: Literature Search Strategy and Data Extraction

One of the biologically plausible mechanisms through which the consumption of dairy products can influence cardiovascular outcomes is by modulating major cardiometabolic risk factors. Therefore, we reviewed the literature on this topic.

We performed a systematic search in PubMed, Embase, Scopus, and Cochrane Library databases for meta-analyses of randomized controlled trials examining the impact of dairy food consumption on the following parameters known to be relevant in relation to cardiovascular risk: body weight and waist circumference, plasma glucose, glycated hemoglobin, insulin resistance (HOMA-IR), blood pressure (systolic and diastolic), plasma lipids (triglycerides, total cholesterol, LDL and HDL cholesterol), and markers of subclinical inflammation (C-reactive protein, TNF-α, IL-6, adiponectin), according to PRISMA (Preferred Reporting Items for Systematic reviews and Meta-Analyses) guidelines [16], up to 30 April 2021. The full details on the search strategy are presented in the Online Supplementary Materials (Supplemental Methods S1). We also performed an additional manual search through the reference lists of original publications to identify further pertinent studies. The search was limited to meta-analyses of human studies and was restricted to papers written in English.

The following inclusion criteria were applied: (1) study design: meta-analyses of randomized controlled trials; (2) subjects: general adult population without prior cardiovascular events; (3) interventions: common dairy foods or dairy supplemented with probiotics. Meta-analyses of randomized controlled trials conducted on populations with special dietary habits (i.e., vegetarians, vegans, child) or with pre-existing cardiovascular diseases and/or cancer were excluded. 

Two investigators (IC and AG) independently reviewed each eligible study, and the following data were extracted: first author’s name, publication year, number of studies, the number and type of participants, intervention, comparison, weighted mean difference (Supplemental Tables S1–S5).

## 3. Results

The results from the literature search and study selection process are shown in Supplemental Figure S1. We identified 36,428 articles (18,666 meta-analyses of prospective cohort studies and 17,762 meta-analyses of RCT) from PubMed, Embase, Scopus, and Cochrane Library databases by 30 April 2021. After two rounds of review and searching citations of retained articles, 332 potentially relevant studies were initially selected (181 meta-analyses of prospective cohort studies and 151 meta-analyses of RCT). After evaluating the full texts, we further excluded 295 studies: 10 for inappropriate study design, 117 for wrong exposure (i.e., milk proteins, vitamin fortified products, etc.), 47 for wrong outcomes (i.e., cerebrovascular diseases, diabetes, etc.), 57 for special population (i.e., pregnant, vegetarians, infant, etc.), 64 inappropriate study setting (breast feeding, animal feeding, pharmacological setting).

### 3.1. Association of Dairy Products with All-Cause Death, CVD or CHD

#### 3.1.1. Total Dairy Foods

The association between globally considered dairy products (i.e., milk, cheese, yogurt) and all-cause death has been investigated by four meta-analyses, two comparing high vs. low dairy intake [17,18] and two exploring the dose–response relationship (Table 1) [19,20]. None of them showed a significant association with all-cause death. As for CVD incidence and mortality, three meta-analyses [19,21,22] are available for the former and one [17] for the latter endpoint. Overall, they indicated that the total consumption of dairy foods was not associated with increased CVD risk, whereas Qin et al. [21] reported a statistically significant 12% reduction in the incidence of CVD for high vs. low dairy intake.

Six meta-analyses have focused on CHD incidence [19,21,22,23,24,25], five of which explored the dose–response relationship [19,22,23,24,25] and consistently showed that a total dairy food consumption up to 200 g per day was not associated with a higher CHD incidence. In one meta-analysis in which dairy intake was quantified in servings/day, rather than g/day, a significant inverse association with CHD incidence emerged for a consumption of three or more servings per day [22]. The only meta-analysis on CHD mortality reported no significant association with dairy consumption [26].

In summation, the available evidence indicates that the consumption of total dairy foods not exceeding 200 g per day is not associated with all-cause deaths, nor with CVD/CHD incidence and mortality. Above this amount, there seems to a trend towards an increased risk of CHD [25].

#### 3.1.2. Full-Fat and Low-Fat Dairy Foods

The only meta-analysis that has explored the consumption of full-fat or low-fat dairy foods in relation to all-cause mortality and CVD incidence found no significant association for consumption of up to 200 g per day of either full-fat or low-fat products (Table 1) [19]. Coherent with this finding, two dose–response meta-analyses reported no association with CHD incidence for the same amount of consumption [19,23]. Two meta-analyses which compared high vs. low intake of full-fat or low-fat products consistently reported no association between full-fat dairy and CHD incidence [21,22], while for low-fat products a significant 10% risk reduction was reported by one of them [22]. A recent meta-analysis on CHD mortality confirmed a neutral association with full-fat or low-fat dairy products [26].

According to the reviewed evidence, consumption of up to 200 g/day of either full-fat or low-fat dairy products is not associated with all-cause death or cardiovascular outcomes in healthy people. For a consumption above this amount the data do not allow a clear trend to be identified.

#### 3.1.3. Milk

Four dose–response meta-analyses that have explored the relationship between milk intake and all-cause death consistently showed a neutral association for a daily consumption of approximately 200 mL (Table 1) [17,19,23,27]. This result is coherent with all but one [23] of the available meta-analyses on milk consumption and CVD outcomes, and with all the meta-analyses on CHD incidence [19,22,23,24,27,28]. The only available meta-analysis on milk consumption and CHD mortality reported a marginal increased risk in the highest versus the lowest category of milk intake, without assessing the dose–response relationship [26].

In a meta-analysis published in 2021, which investigated the association of low-fat and full-fat milk consumption with fatal and non-fatal CHD events, Jakobsen et al. [28] showed a neutral relationship for a daily consumption of up to 200 g of low-fat milk, but a significant 8% higher risk associated with the same amount of full-fat milk (RR: 1.08, 95% CI: 1.00–1.16). More recent studies—not included in this meta-analysis—in which the different fat content in milk was evaluated in relation to death from all-cause, CVD or CHD, showed no significant association with any of the considered endpoints irrespective of the fat content of milk [34,35,36].

Overall, the evidence indicates that for milk consumption the association with all-cause mortality and cardiovascular endpoints is neutral for a daily intake up to 200 mL. Above this quantity, data are too scanty to allow a meaningful evaluation of a trend.

#### 3.1.4. Fermented Dairy Foods, Cheese and Yogurt

In a recent dose–response meta-analysis [19] the consumption of fermented dairy products (i.e., sour milk products, yogurt, cheese) was associated with a marginal, but statistically significant, risk reduction (−2%) of all-cause mortality and CVD incidence for each 20 g increase in fermented dairy consumption (Table 1).

This finding was confirmed by a later meta-analysis, which showed a significant 20% lower risk of total CV events [29]. As for fatal CVD, the only available meta-analysis found no significant association with fermented dairy intake [29]. Similar results were reported in the two available meta-analyses on CHD incidence [19,29]. It is, however, notable that in the paper by Zhang et al. [29], high vs. low consumption of fermented dairy was associated with a significant 18% lower risk of myocardial infarction (MI). An inverse association between fermented dairy intake and MI was also reported by some recently published prospective studies not included in the published meta-analyses [37,38].

Cheese and yogurt, the two main components of the fermented dairy food group, were also analyzed. Cheese consumption was not associated with all-cause mortality [17,19,30]; for CVD incidence, either a neutral association [22] or a statistically significant 10 and 13% risk reduction [29,31] were reported for high vs. low cheese intake, whereas one out of two available dose–response analyses [19,31] showed a marginal but significant 2% risk reduction associated with the consumption of 10 g of cheese per day [19]. No relationship with CVD mortality was reported [17]. A daily amount of 50 g of cheese (a standard serving of hard and semi-hard types) was associated with a statistically significant 10 and 14% lower risk of CHD in two meta-analyses including a large number of subjects [22,31]. This finding is shared by the analyses comparing high vs. low cheese intakes, but for lower amounts of cheese consumption the magnitude of the risk reduction is smaller (i.e., −4% for 20 g per day) or null (i.e., for 10 g per day) [19,28]. Similarly, there is no association with CV endpoints for higher intakes [31].

With regard to yogurt, the consumption of 200 g per day was associated with a significant 5% lower total mortality and with an 8% risk reduction of total CVD events [32,33]. A further confirmation of the inverse relationship between a high yogurt consumption and CVD comes from a recent meta-analysis of 10 cohort studies conducted by Zhang et al. [29] that found a statistically significant 22% lower risk of CV events in the high vs. low consumption group.

These findings expand prior knowledge provided by a dose–response analysis, which reported no association between yogurt–but at a lower amount of consumption (i.e., 50 g)—and all-cause death or CVD events [19]. As for CHD incidence, data from five meta-analyses consistently showed a neutral relationship with yogurt consumption [19,21,22,28,33]; this has been confirmed by Jakobsen et al. [28] for both low-fat and full-fat yogurt.

In summation, the available evidence indicates that the consumption of fermented dairy foods is inversely associated with all-cause death and CVD risk. In particular, a generous serving of yogurt (≥200 g per day) is associated with a lower CVD risk, while a moderate cheese consumption (50 g/day) is associated with a reduced risk of CHD.

### 3.2. Effects of Dairy Foods on Cardiovascular Risk Factors

Among the mechanisms by which the consumption of dairy products can influence cardiovascular outcomes, the modulation of the CV risk factors profile may certainly play a relevant role. Since dairy products have different nutrients composition and a different food matrix, they may also have different effects on major CV risk factors. Here, we review the evidence from meta-analyses of clinical trials relative to body weight and body fat distribution, fasting glucose and glycated hemoglobin, insulin resistance, blood pressure, plasma lipids, and markers of subclinical inflammation (Table 2).

#### 3.2.1. Body Weight/Waist Circumference

Several meta-analyses have focused on the effect of total dairy on body weight [39,40,41,42,43,44,45]. A marginal weight reduction ranging from 0.6 to 1.2 kg was reported in some meta-analyses in which dairy supplementation was implemented within the context of an energy-restricted diet [40,44,45], whereas a neutral effect or a marginal weight increase—ranging from 0.36 to 0.60 kg—was shown by studies where dairy supplementation was not associated with energy restriction (Supplemental Table S1) [40,43,44]. The effect of low-fat or full-fat dairy foods on body weight was explored by Benatar et al. [43] in a meta-analysis showing a statistically significant increase for either low-fat or full-fat dairy products in the context of a dietary regimen without energy restriction. No information is available on the effects of individual foods such as cheese, milk, or yogurt on body weight and fat distribution.

The relation of total dairy consumption with waist circumference was investigated by four meta-analyses [39,40,43,44], three of which show a small, statistically significant reduction in waist circumference, ranging from −2.43 to −1.09 cm [39,40,44]; in these meta-analyses, dairy supplementation was implemented within the context of energy-restricted diets. As for dose–effect, Geng et al. [40] reported a non-significant effect for a supplement of 0.5 to 5.24 g/day of total dairy when evaluated within the context of a diet with or without energy restriction.

The effect of low-fat or full-fat dairy consumption on waist circumference has been explored by Benatar et al. [43] who report no significant association; no data are available to discern the effects of individual foods (i.e., cheese, milk, yogurt). Interventions including the consumption of dairy products supplemented with probiotics were associated with a marginal, but statistically significant, reduction in waist circumference [46].

Overall, the available evidence indicates that the effect of dairy foods on weight/waist circumference is marginal and largely driven by the energy content of the diet.

#### 3.2.2. Fasting Glucose/Glycated Hemoglobin

The search retrieved four meta-analyses on the effects of dairy food consumption on fasting glucose (Supplemental Table S2) [43,46,47,48]. O’Connor et al. [48] reported a marginal but statistically significant increase in fasting glucose (1.3 mg/dL) with a high total dairy intake. When full-fat or low-fat products were analyzed separately, a marginal increase in fasting glucose was reported for full-fat dairy [43]. The two meta-analyses dealing with low-fat dairy provided conflicting findings: one reports a neutral effect [43], whereas the other shows a modest, but significant increase of 0.07 mmol/L (i.e., 1.26 mg/dL) [48]. Somewhat at variance with this latter observation, however, in a meta-analysis of only four studies the same author reported marginally lower glycated hemoglobin for high versus low consumption of total dairy (−0.09%; 95% CI: −0.16, −0.03) [48]; the finding was not confirmed when only low-fat products were analyzed.

As for specific dairy foods, no significant effect on glucose was reported for cheese consumption, whereas a marginal increase (1.44 mg/dL; 95% CI: 0.36, 2.52) was reported for a high consumption of milk/and or yogurt [48]; again, at variance with this finding, in an analysis of three studies the same author reports a lower value of glycated hemoglobin associated with higher milk and/or yogurt consumption.

Two recent meta-analyses have focused on the relationship of yogurt and other dairy foods enriched with probiotics with blood glucose, showing a moderate reduction ranging from 6 to 13 mg/dL [46,47]. Dixon et al. [47] have explored the effect of yogurt or milk enriched with probiotics: a significant reduction of 12.88 mg/dL in fasting glucose in the intervention group vs. placebo was reported for yogurt, but not for milk; they also showed a modest decrease (−0.55%; 95% CI: −1.03, −0.06) or no change in glycated hemoglobin after yogurt or milk intake, respectively, thus suggesting that probiotics might exert some beneficial effects on glucose homeostasis.

In summation, the evidence for a relationship between the consumption of dairy foods—total dairy, full-fat or low-fat products, or specific items such as cheese or milk—and glucose/glycated hemoglobin is weak; instead, fermented products added with probiotics might be beneficial.

#### 3.2.3. Insulin/Insulin Resistance

Three meta-analyses have explored the relation of total dairy intake with insulin resistance as estimated by the HOMA index [39,43,48]; only one reports a significant improvement in insulin sensitivity associated with intervention of dairy supplementation with or without an energy deficit or caloric restriction (Supplemental Table S2) [39]. In this same meta-analysis, marginal reductions in waist circumference and weight were also reported, therefore it is not straightforward to extrapolate to what extent the improved HOMA-IR is mediated by changes in body weight or by the dietary intervention per se.

The effect of specific dairy food on insulin sensitivity has been extensively studied by O’Connor et al. [48] who have meta-analyzed the few available RCTs dealing with low-fat products (four studies), cheese (two studies), milk and yogurt (six studies), fermented dairy products (four studies), without finding any significant associations.

In summation, the available evidence is scant and overall does not support an association of dairy foods consumption with amelioration of insulin sensitivity.

#### 3.2.4. Blood Pressure

Benatar et al. [43] have meta-analyzed the few studies on the effects of dairy intake on blood pressure; no significant effect was found for either total dairy or low- or full-fat products (Supplemental Table S3). The other available studies concern yogurt and dairy foods enriched with probiotics [47,49,50]. In particular, in a meta-analysis of 15 studies Usinger et al. [49] found a significant reduction in systolic blood pressure (−2.45 mmHg) in the group assigned to fermented milk consumption, whereas in a meta-analysis of three studies, Dixon et al. [47] found a reduction of 3.54 mmHg in diastolic blood pressure, associated with increased consumption of yogurt enriched with probiotics.

On the overall there is no evidence for a detrimental effect of dairy consumption on blood pressure; if anything, a slight improvement with increasing consumption of yogurt enriched with probiotics and fermented milk has been described.

#### 3.2.5. Plasma Lipids

Dairy foods have long been considered unnecessary sources of saturated fats with adverse effects on plasm lipids; however, this concept is based on old studies and needs to be reconsidered in the light of the currently available evidence. The effects of dairy foods on plasma lipids have been extensively studied; data are available for total cholesterol, LDL cholesterol, HDL cholesterol and triglycerides (Supplemental Table S4).

With regard to total cholesterol, no detrimental effects were reported in a network meta-analysis in which total dairy foods were compared with fish, red meat, or sugar-sweetened beverages (SSBs) [51]. As for specific foods, the effect of cheese on total cholesterol was explored by one meta-analysis which shows an average reduction of 0.28 mmol/L (i.e., 10 mg/dL) for hard cheese intake vs. butter intake [52]. Seven meta-analyses of RCTs on the effects of yogurt and other dairy products enriched with probiotics consistently show a reduction in total cholesterol ranging from 6.8 to 16 mg/dL [46,47,53,54,55,56,57]. 

For LDL cholesterol, no significant effects were reported in a meta-analysis in which total dairy foods were compared with fish, red meat, or SSBs [51]. The only meta-analysis exploring the separate effect of full-fat or low-fat products showed a neutral effect for both [43]. The effects of cheese on plasma cholesterol were studied in one meta-analysis of five studies in which the intake of hard cheese was compared with that of butter with a similar polyunsaturated/saturated fatty acids ratio; an average reduction of 0.22 mmol/L (i.e., 7.9 mg/dL) was reported for LDL cholesterol in the cheese group [52].

Seven meta-analyses have focused on dairy products enriched with probiotics [46,47,53,54,55,56,57]; the results consistently show a reduction in LDL cholesterol ranging from 7.6 to 18 mg/dL. Information on the dose is not available; the few existing data indicate a significant effect for a daily consumption of 80–600 mL of probiotic fermented milk [53].

The findings are very similar for HDL cholesterol. A neutral effect is reported for total, low-, or full-fat dairy, whereas de Goede et al. [52] report a reduction in HDL cholesterol of 0.05 mmol/L (i.e., 0.9 mg/dL) when hard cheese was compared with butter; this is in parallel with the reduction in total cholesterol and LDL cholesterol described in the same study. As for products enriched with probiotics, five out of six available meta-analyses show a neutral effect on HDL cholesterol [47,53,54,55,56]. The only exception is a meta-analysis of four RCTs showing that dairy foods enriched with probiotic increased HDL cholesterol by 0.26 mmol/L (i.e., 4.7 mg/dL) [46]; it is of note that in this same study a reduction in LDL cholesterol of 0.5 mmol/L (i.e., 18 mg/dL) was also reported; no data are available on the dosage.

Several meta-analyses have explored the effect of dairy consumption on plasma triglycerides [46,47,51,52,55,56]. No significant effect was reported by a meta-analysis in which dairy consumption was evaluated in comparison with fish, red meat, or SSBs [51]. The only available meta-analysis focusing on cheese shows a neutral effect [52]. Four meta-analyses have focused on dairy enriched with probiotics; three show a neutral effect [47,55,56], whereas a significant reduction of 0.46 mmol/L (8.3 mg/dL) was reported by Companys et al. [46] in a meta-analysis of three studies. In this same work, as already mentioned, a significant reduction in LDL cholesterol and a significant increase in HDL cholesterol was also reported.

Overall, the available evidence does not support a detrimental effect of dairy consumption on plasma lipids; conversely, there are consistent indications that consumption of dairy enriched with probiotics can ameliorate the plasma lipids profile and, in particular, reduce total and LDL cholesterol in people with hypercholesterolemia.

#### 3.2.6. Subclinical Inflammation

Two meta-analyses have explored the effects on subclinical inflammation of total dairy with C-reactive protein (Supplemental Table S5) [43,58]. While Benatar et al. [43] have shown a neutral effect for total dairy and full-fat or low-fat products, a very recent meta-analysis based on a larger number of studies showed that a high total dairy intake could significantly reduce C-reactive protein (−0.24 mg/L) as well as other markers of subclinical inflammation such as TNF-α (−0.66 pg/mL) and IL-6 (−0.74 pg/mL) and increase serum adiponectin levels (+2.42 μg/mL), a cytokine with anti-inflammatory properties [58].

As for specific dairy foods, data are available only for milk and yogurt; for these products, a high vs. low consumption has a neutral effect on C-reactive protein and IL-6 and a reducing effect on TNF-α (−0.37 pg/mL), while increasing serum adiponectin levels (+14.28 μg/mL) [58].

Overall the evidence is scant, but suggestive of a beneficial effect of specific food items (yogurt and milk) on markers of subclinical inflammation.

## 4. Discussion

The meta-analyses of cohort studies reviewed here do not support a detrimental relation of dairy foods consumption with total mortality or cardiovascular diseases. Indeed, there is consistent evidence that a total consumption of dairy foods (i.e., including milk, cheese, and yogurt) up to 200 g per day has a neutral association with CVD risk, independent of whether full-fat or low-fat products are considered. As for specific dairy foods, a neutral association was found for milk, while fermented products—cheese and yogurt—were associated with a lower risk of total mortality and CV events. These results confirm and expand our previous observations on the relevance of food choices with regard to cardiovascular health [13], and highlight the wide heterogeneity existing among dairy foods with regard to their association with CVD [59]. This complexity is partly due to potential interrelated influences of different nutrients and non-nutrient bioactive compounds, as well as other food characteristics (i.e., fermentation physical features, processing and cooking procedures) which can modulate the bioavailability and the metabolic effects of nutrients and, consequently, their impact on health [60]. By focusing on foods rather than on nutrients (namely SFAs) the present work is aligned with the most recent research on nutrition and health, which emphasizes food choices and dietary patterns above the nutrients composition of the diet as major determinants of health [13,61]. This approach is also more suitable for the translation of the information into clinical recommendations, as it is more understandable to lay people.

Current nutritional recommendations for CVD prevention in adults are mainly informed by the evidence that SFAs, largely present in dairy, contribute to increasing plasma cholesterol levels which, in turn, increases CV risk; however, recent data indicate that not all SFAs have the same metabolic effects [62,63,64].

The knowledge that different sources of SFAs have a different impact on CVD is not new. Early prospective studies indicated that the consumption of dairy fat (mainly milk and butter) was associated with an increased mortality from CHD [65,66,67]; however, when cheese was included among the sources of dairy fat, the correlation coefficients were reduced and become less statistically significant [67].

A subsequent study based on quantities of 40 food items available for consumption from the 1977 Food Balance Sheets (FBSs) of the Food and Agriculture Organization (FAO), relative to 40 countries, has investigated the relationship between a dietary lipid score that combined the intakes of cholesterol and saturated fat (Cholesterol–Saturated Fat Index, CSI) and CHD mortality [68]. The results showed that in Finland CHD mortality was three to five times higher than in France, even though the CSIs of these two countries were almost the same; however, the quality of products consumed was different, since milk intake was 3.5 times greater in Finland than in France [68].

The difference between milk and cheese in relation to CVD outcomes has been ascribed, among others, to the higher calcium content of cheese. On one hand, calcium might partly limit the absorption of SFAs [67,69]; on the other hand, extracellular and intracellular calcium concentrations influence cell membrane potentials of excitable tissues, including the myocardium. In vitro and in silico studies have shown that cardiomyocyte calcium handling is a major determinant of excitation–contraction coupling [70]. However, the clinical implications of these findings remain unclear. Results of human studies on the role of calcium in CV health are incoherent: while dietary calcium does not seem associated with the incidence of CVD, calcium supplementation is reported to increase the risk of MI [71].

There is evidence that differences in the chain length of SFAs lead to different physicochemical properties and biological effects [72]. The physical and nutritional structure of foods can further modulate their biological effects by influencing the digestion, absorption, and bioavailability of the various nutrients. This is particularly true for a complex food group such as dairy foods. For example, despite their high content in long-chain saturated fatty acids (60% of dairy fat), dairy products are an important source of potentially beneficial compounds, such as medium- and odd-chain saturated fats, natural trans fats, unsaturated and branched-chain fats, branched amino acids, vitamin K1 and K2, and calcium [72]. Probiotics and bioactive compounds naturally contained in fermented dairy further increase the complexity of this food group, since their presence can influence the composition and function of the gut microbiota of the host, which in turn modulates the cardiometabolic risk [64]. In more detail, the probiotics’ activity favors the intestinal epithelial integrity and reduces the low-grade inflammation due to metabolic endotoxemia; moreover, it modulates host microbiota composition, thus improving the energy homeostasis, the intermediate metabolism, and the insulin sensitivity [72,73].

The relationship between the various categories of dairy products and cardiovascular disease is largely, though not completely, mediated by their impact on major CV risk factors. To further substantiate the findings on hard CV outcomes (i.e., fatal and non-fatal events), and investigate biologically plausible mechanisms through which dairy may impact on CVD risk, we have reviewed the available meta-analyses of RCTs exploring the effects of the various dairy foods (i.e., low/full-fat dairy, milk, cheese, yogurt) on major CV risk factors, namely: body weight/waist circumference, plasma glucose/glycated hemoglobin, insulin sensitivity, blood pressure, plasma lipids, subclinical inflammation.

For body weight/waist circumference, either total dairy foods or specific items showed a substantially neutral effect; very modest changes in weight or waist circumference were reported in some meta-analyses, but they were mainly driven by the energy content of the dietary intervention. In more detail, when the dietary intervention is performed without limitation in the energy intake, enhanced dairy consumption may lead to increased energy intake and weight gain [40,44,45]. Conversely, dairy products may have modest effects in facilitating weight loss in the context of an energy-restricted diet [40,41,44,45] and there is some suggestion that yogurt may be protective against long-term weight gain [72].

The potential mechanisms through which dairy products may affect body weight include a reduction in appetite due to a higher intake of proteins, particularly casein, which has a good satiating and thermogenic effect [60,74,75]. Furthermore, the increase in calcium intake may be beneficial for weight loss because it can reduce fatty acids absorption and lipogenesis and stimulate lipolysis, by decreasing plasma calcitriol [1,25 (OH)2 D] levels and parathyroid hormone or calciotropic hormones synthesis [76].

A marginal or null effect of total or specific dairy foods has been reported on fasting glucose and glycated hemoglobin in population without diabetes. Dairy products contain variable amounts of sugar, which may explain the marginal and non-clinically relevant increase in fasting glucose reported by O’Connor et al. [48]; however, no information about the sugar content is provided. Furthermore, it not possible to estimate the confounding effect on plasma glucose of other foods consumed in association with dairy products. Conversely, there are some indications that fermented dairy products added with probiotics might exert beneficial effects on fasting glucose and glycated hemoglobin. This is in line with the protective effect of yogurt consumption on the risk of developing type 2 diabetes reported by some authors [60,77].

The evidence regarding insulin sensitivity and subclinical inflammation is scant and overall does not support a significant impact of this food group.

The effects of dairy on blood pressure have also been shown to be substantially neutral; notwithstanding, several studies have provided evidence for biologically plausible mechanisms through which dairy foods might lower blood pressure (i.e., calcium, vitamin D, potassium, and phosphorous content, bioactive small peptides effect, and probiotic activity) [78]. Moreover, habitual dairy intake is associated with lower blood pressure levels in cross-sectional observational studies. Additionally, in the large randomized DASH trial dairy intake was associated with lower blood pressure; however, the dietary intervention included multiple dietary components besides low-fat dairy food (i.e., reduced total and saturated fat intake and increased consumption of vegetables and fruit), and therefore it is not possible in this study to estimate the effects of each single intervention [79].

The effects of dairy foods on plasma lipids have been extensively studied. The well-established knowledge that saturated fats in the diet increase serum cholesterol, which in turn leads to an increased risk of CVD, was the basis on which over time, guidelines for CVD prevention in the general adult population focused on the control of plasma LDL cholesterol, to be achieved, among others, by limiting dairy foods consumption and by substituting whole fat with low-fat products [80]. Indeed, the evidence reviewed here does not support an adverse effect of moderate dairy food consumption (either low or full fat) on plasma lipids, and the literature on this topic is constantly growing. Notably, despite dairy fat consists of around 60% SFAs, they represent a mixture of various subtypes, with different effects on the lipid profile. For instance, besides long-chain SFAs with LDL-raising effect as lauric acid, myristic acid, palmitic acid and stearic acid, dairy fatty acids include (9.8%) medium-chain saturated fatty acids (MCSFAs) (between 6 to 12 carbons, i.e. 6:0 to 12:0), (31.9%) odd-chain SFAs (OCSFAs) (15:0, 17:0), (25%) monounsaturated and (2.3%) polyunsaturated fatty acids (18:1n-9, 18:2n-6 and 18:3n-3), branched-chain saturated fatty acids, and trace amounts of natural (ruminant) trans fats (i.e., trans-palmitoleic acid, 156–158 trans-16:1n-7) [81,82].

Accordingly, a meta-analysis of five randomized controlled trials from Denmark, Norway, and Australia supports heterogeneous effects of dairy on lipid profiles depending on the type of food consumed [52]. Indeed, in this paper, hard cheese compared to butter lowers total cholesterol by 5% and LDL cholesterol by approximately 6.5% despite a similar ratio of polyunsaturated/saturated fatty acids (P/S ratio); this indicates that the different effects of cheese and butter on plasma lipid levels in this meta-analysis cannot be due to the relative amounts of SFAs and PUFAs in the two diets. More probably, the different responses should be attributed to other dairy components or to the specific processing methods utilized for butter and cheese production.

In this regard, it is notable that dairy products enriched with probiotics have a clear hypocholesterolemic effect, since they can modify the gut flora by promoting bacteria strains able to ferment dietary fiber (non-digestible carbohydrates) from seeds, wholegrains, legumes, and vegetables, which are not metabolized or absorbed while passing through the upper gastrointestinal tract. Fiber fermentation leads to the production of short-chain fatty acids (SCFAs) that exert local and systemic effects, such as the inhibition of the hepatic cholesterol synthesis and the stimulation of liver cholesterol uptake [83].

Furthermore, SCFAs, especially butyrate, can lower the plasma levels of pro-inflammatory markers (high-sensitivity C-reactive protein, TNF-α and IL-6) by acting at the gene expression level or by the activation of the MAPK pathway [84].

In summary, by combining data on CV events and risk factors, this study provides coherent evidence for a neutral effect of a moderate consumption of total dairy food on CVD irrespective of fat content. A beneficial effect of some specific items (i.e., fermented products and products added with probiotics), largely mediated through their effect on major CV risk factors—mainly lipids and subclinical inflammation—also emerges.

## 5. Conclusions

This study highlights the complexity of the relationship between different dairy foods and cardiovascular diseases as well as their risk factors. Altogether, the results indicate that the association of dairy intake with cardiovascular risk is largely driven by the food type (i.e., cheese, yogurt, milk). These findings may inform dietary recommendations for CVD prevention by allowing healthy people with normal plasma cholesterol levels a more liberal consumption of up to 200 g/day of total dairy foods (including milk, cheese, and yogurt), irrespective of being full or low fat. Within this amount of consumption, fermented dairy should be preferred (i.e., one generous serving/day of yogurt or three small servings/week of cheese).

Further studies should be undertaken to clarify the mechanisms of the beneficial impact of probiotics and other components of dairy foods such as whey protein and calcium on the cardiovascular risk-factor profile.

## Figures and Tables

**Table 1:** Summary of the available meta-analyses of prospective cohort studies on the relation between dairy products and CV hard endpoints or all-cause mortality.

	Meta-Analysis	Neutral Relation	Inverse Relation (% Risk Reduction)	Positive Relation (% Risk Increase)
**TOTAL DAIRY**				
**All-cause mortality**	O’Sullivan 2013 [17]	✓		
	Guo 2017 [19]	✓		
	Eleftheriou 2018 [18]	✓		
	Schwingshackl 2018 [20]	✓		
**CVD incidence**	Qin 2015 [21]		✓ (−12% high vs. low intake)	
	Alexander 2016 [22]	✓		
	Guo 2017 [19]	✓		
**CVD mortality**	O’ Sullivan 2013 [17]	✓		
**CHD incidence**	Soedamah-Muthu 2011 [23]	✓		
	Qin 2015 [21]	✓		
	Alexander 2016 [22]	✓ (high vs. low intake)	✓ (−14% per > 3 s/d)	
	Guo 2017 [19]	✓		
	Soedamah-Muthu 2018 [24]	✓		
	Bechthold 2019 [25]	✓		
**CHD mortality**	Mazidi 2019 [26]	✓		
**FULL-FAT DAIRY**				
**All-cause mortality**	Guo 2017 [19]	✓		
**CVD incidence**	Guo 2017 [19]	✓		
**CHD incidence**	Soedamah-Muthu 2011 [23]	✓		
	Qin 2015 [21]	✓		
	Alexander 2016 [22]	✓		
	Guo 2017 [19]	✓		
**CHD mortality**	Mazidi 2019 [26]	✓		
**LOW-FAT DAIRY**				
**All-cause mortality**	Guo 2017 [19]	✓		
**CVD incidence**	Guo 2017 [19]	✓		
**CHD incidence**	Soedamah-Muthu 2011 [23]	✓		
	Qin 2015 [21]	✓		
	Alexander 2016 [22]		✓ (−10% high vs. low intake)	
	Guo 2017 [19]	✓		
**MILK**				
**All-cause mortality**	Soedamah-Muthu 2011 [23]	✓		
	O’Sullivan 2013 [17]	✓		
	Mullie 2016 [27]	✓		
	Guo 2017 [19]	✓		
**CVD incidence**	Soedamah-Muthu 2011 [23]		✓ (−6% per 200 mL/d)	
	Alexander 2016 [22]	✓		
	Guo 2017 [19]	✓		
**CVD mortality**	O’Sullivan 2013 [17]	✓		
**CHD incidence**	Soedamah-Muthu 2011 [23]	✓		
	Alexander 2016 [22]	✓		
	Mullie 2016 [27]	✓		
	Guo 2017 [19]	✓		
	Soedamah-Muthu 2018 [24]	✓		
	Jakobsen 2021 [28]	✓		
**CHD mortality**	Mazidi 2019 [26]			✓ (+4% high vs. low intake)
**FERMENTED DAIRY PRODUCTS**				
**All-cause mortality**	Guo 2017 [19]		✓ (−2% per 20 g/d)	
**CVD incidence**	Guo 2017 [19]		✓ (−2% per 20 g/d)	
	Zhang 2020 [29]		✓ (−20% high vs. low intake)	
**CVD mortality**	Zhang 2020 [29]	✓		
**CHD incidence**	Guo 2017 [19]	✓		
	Zhang 2020 [29]	✓		
**CHEESE**				
**All-cause mortality**	O’Sullivan 2013 [17]	✓		
	Guo 2017 [19]	✓		
	Tong 2017 [30]	✓		
**CVD incidence**	Alexander 2016 [22]	✓		
	Chen 2017 [31]	✓ per 50 g/d	✓ (−10% high vs. low intake)	
	Guo 2017 [19]		✓ (−2% per 10 g/d)	
	Zhang 2020 [29]		✓ (−13% high vs. low intake)	
**CVD mortality**	O’Sullivan 2013 [17]	✓		
**CHD incidence**	Qin 2015 [21]		✓ (−16% high vs. low intake)	
	Alexander 2016 [22]		✓ (−14% per 50 g/d)	
	Chen 2017 [31]		✓ (−10% per 50 g/d)	
	Guo 2017 [19]	✓		
	Jakobsen 2021 [28]		✓ (−4% per 20 g/d)	
**YOGURT**				
**All-cause mortality**	Guo 2017 [19]	✓		
	Gao 2020 [32]	✓ high vs. low intake	✓ (−5% per 200 g/d)	
**CVD incidence**	Alexander 2016 [22]	✓		
	Guo 2017 [19]	✓		
	Wu 2017 [33]	✓ high vs. low intake	✓ (−8% per ≥ 200 g/d)	
	Zhang 2020 [29]		✓ (−22% high vs. low intake)	
**CVD mortality**	Gao 2020 [32]	✓ high vs. low intake	✓ (−8% per 200 g/d)	
**CHD incidence**	Qin 2015 [21]	✓		
	Alexander 2016 [22]	✓		
	Wu 2017 [33]	✓		
	Guo 2017 [19]	✓		
	Jakobsen 2021 [28]	✓		

**Table 2:** Summary of the available meta-analyses of randomized controlled trials on the effect of dairy products on cardiovascular risk factors.

	No. of Meta-Analyses Reporting No Effect	No. of Meta-Analyses Reporting a Significant Reduction	No. of Meta-Analyses Reporting a Significant Increase
**TOTAL DAIRY**			
**Body weight**	3	5 *	2
**Waist circumference**	3	3	0
**Fasting glucose**	0	0	2
**Glycated hemoglobin**	0	1	0
**Insulin resistance (HOMA-IR)**	2	1	0
**Systolic blood pressure**	1	0	0
**Diastolic blood pressure**	1	0	0
**Total cholesterol**	1	0	0
**LDL cholesterol**	2	0	0
**HDL cholesterol**	1	0	0
**Triglycerides**	1	0	0
**C-reactive protein**	1	1	0
**TNF-α**	0	1	0
**IL-6**	0	1	0
**Adiponectin**	0	0	1
**FULL-FAT DAIRY PRODUCTS**			
**Body weight**	0	0	1
**Waist circumference**	1	0	0
**Fasting glucose**	0	0	1
**Systolic blood pressure**	1	0	0
**Diastolic blood pressure**	1	0	0
**LDL cholesterol**	1	0	0
**HDL cholesterol**	1	0	0
**C-reactive protein**	1	0	0
**LOW-FAT DAIRY PRODUCTS**			
**Body weight**	0	0	1
**Waist circumference**	1	0	0
**Fasting glucose**	1	0	1
**Glycated hemoglobin**	1	0	0
**Insulin resistance (HOMA-IR)**	1	0	0
**Systolic blood pressure**	1	0	0
**Diastolic blood pressure**	1	0	0
**LDL cholesterol**	1	0	0
**HDL cholesterol**	1	0	0
**C-reactive protein**	1	0	0
**MILK AND/OR YOGURT**			
**Fasting glucose**	0	0	1
**Glycated hemoglobin**	0	1	0
**Insulin resistance (HOMA-IR)**	1	0	0
**C-reactive protein**	1	0	0
**TNF-α**	0	1	0
**IL-6**	1	0	0
**Adiponectin**	0	0	1
**CHEESE**			
**Fasting glucose**	1	0	0
**Insulin resistance (HOMA-IR)**	1	0	0
**Total cholesterol**	0	1	0
**LDL cholesterol**	0	1	0
**HDL cholesterol**	0	1	0
**Triglycerides**	1	0	0
**FERMENTED DAIRIES OR DAIRIES PLUS PROBIOTICS**			
**Waist circumference**	0	1	0
**Fasting glucose**	2	2	0
**Glycated hemoglobin**	1	1	0
**Insulin resistance (HOMA-IR)**	1	0	0
**Systolic blood pressure**	2	1	0
**Diastolic blood pressure**	3	1	0
**Total cholesterol**	0	7	0
**LDL cholesterol**	0	7	0
**HDL cholesterol**	5	0	1
**Triglycerides**	3	1	0

* 4 meta-analyses including studies where dairy supplementation was associated with energy restriction; 1 meta-analysis including studies where dairy supplementation was not associated with energy restriction. CV: cardiovascular.

## Data Availability

Not applicable.

## Supplementary Materials

The following are available online at https://www.mdpi.com/article/10.3390/nu14040831/s1: Supplemental Methods S1: Search strategy. Supplemental Figure S1: PRISMA flow diagram. Table S1: Summary of meta-analyses of randomized controlled trials on the effect of dairy products intake on body weight and waist circumference. Table S2: Summary of meta-analyses of randomized controlled trials on the effect of dairy products intake on fasting glucose, glycated hemoglobin and insulin resistance. Table S3: Summary of meta-analyses of randomized controlled trials on the effect of dairy products intake on blood pressure. Table S4: Summary of meta-analyses of randomized controlled trials on the effect of dairy products intake on plasma lipids. Table S5: Summary of meta-analyses of randomized controlled trials on the effect of dairy products intake on markers of subclinical inflammation.
