# Supplementary material for: Consumption of Dairy Foods and Cardiovascular Disease: A Systematic Review
Source: Nutrients. 2022 Feb 16;14(4):831. doi: 10.3390/nu14040831 (PMC8875110; doi:10.3390/nu14040831)
Supplement: Supplementary file 1 [file nutrients-14-00831-s001.zip › nutrients-1552871-supplementary.pdf]

Supplementary Material

CONTENTS

Supplemental Methods S1. Search strategy

Supplemental Figure S1. PRISMA flow diagram

Supplemental Table S1. Summary of meta-analyses of randomized controlled trials on the effect of dairy products intake on body weight and waist circumference.

Supplemental Table S2. Summary of meta-analyses of randomized controlled trials on the effect of dairy products intake on fasting glucose, glycated hemoglobin and insulin resistance**.**

Supplemental Table S3. Summary of meta-analyses of randomized controlled trials on the effect of dairy products intake on blood pressure.

Supplemental Table S4. Summary of meta-analyses of randomized controlled trials on the effect of dairy products intake on plasma lipids.

Supplemental Table S5. Summary of meta-analyses of randomized controlled trials on the effect of dairy products intake on markers of subclinical inflammation.

Supplemental Methods S1. Search strategy

Search terms for meta-analysis of prospective cohort studies:

(dairy OR dairy products OR total dairy OR full fat dairy OR low fat dairy OR milk OR fermented dairy OR cheese OR yogurt) AND (all-cause mortality OR coronary heart disease incidence OR coronary heart disease mortality OR cardiovascular disease incidence OR cardiovascular disease mortality OR coronary artery disease OR acute myocardial infarction)

Search terms for meta-analysis of RCT:

(dairy OR dairy products OR total dairy OR full fat dairy OR low fat dairy OR milk OR fermented dairy OR cheese OR yogurt) AND (body weight OR weight gain OR weight reduction OR waist circumference OR blood pressure OR systolic blood pressure OR diastolic blood pressure OR glycemia OR fasting glucose OR plasma glucose OR blood glucose OR glycated hemoglobin OR HbA1c OR HOMA index OR insulin resistance OR insulin sensitivity OR blood lipids OR cholesterol OR total cholesterol OR LDL cholesterol OR HDL cholesterol OR triglycerides OR inflammation OR C reactive protein OR CRP OR interleukin 6 OR IL 6 OR tumor necrosis factor alpha OR TNF alpha OR adiponectin)

Filters applied:

Meta-analysis; Humans; English.

Supplemental Figure S1. PRISMA flow diagram

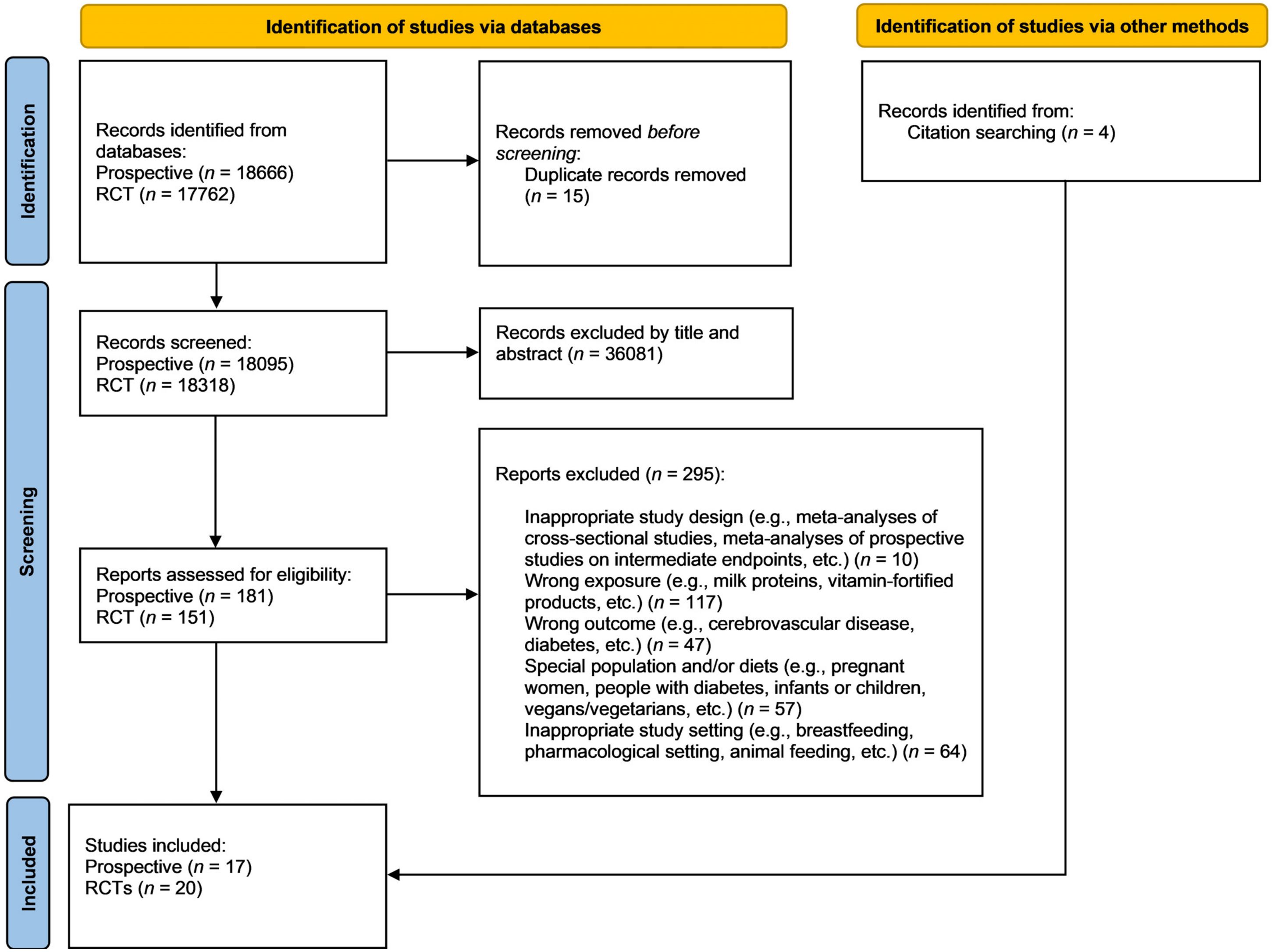

From: Page MJ, McKenzie JE, Bossuyt PM, Boutron I, Hoffmann TC, Mulrow CD, et al. The PRISMA 2020 statement: an updated guideline for reporting systematic reviews. BMJ 2021;372:n71. doi:10.1136/bmj.n71.

Supplemental Table S1. Summary of meta-analyses of randomized controlled trials on the effect of dairy products intake on body weight and waist circumference.

**Body weight**						
**DAIRY PRODUCT**	**Author, year**	**No. of studies**	**No. and type of subjects**	**Intervention (duration and dose)**	**Comparison**	**Weighted Mean Difference (95% CI)**
	**Abargouei 2012**	5	453 Not defined	21-48 wk Increased dairy (3-4 s/d) without energy restriction	Intervention vs. usual diet	0.33 (–0.35; 1.00) kg
	**Abargouei 2012**	9	430 Not defined	8-48 wk Increased dairy (3-5 s/d) with energy restriction	Intervention vs. usual diet	–1.29 (–1.98; –0.60) kg
	**Chen 2012**	13	1668 with normal weight, overweight, obesity	1-36 mo 1-6.5 s/d of dairy without energy restriction	Intervention vs. usual diet (without energy restriction)	0.39 (–0.36; 1.13) kg
**TOTAL DAIRY**	**Chen 2012**	16	850 with normal weight, overweight, obesity	2-12 mo 1-6.5 s/d of dairy with energy restriction	Intervention vs. usual diet (with energy restriction)	–0.79 (–1.35; –0.23) kg
	**Benatar 2013**	18	1629 with normal weight, overweight, obesity, MS (BMI: 25.6±6.2)	10-39 wk (median 26 wk) Increased dairy food without additional interventions	Intervention vs. usual diet	0.60 (0.30; 0.90) kg
	**Booth 2015**	31	2091 with normal weight, overweight, obesity	3-24 mo (median 7 mo) Calcium intake by 482–2400 mg/d via dairy products (low fat, no fat	Intervention vs. usual diet or moderate dairy or low–dairy or low–Ca diet	–0.06 (–0.54; 0.43) kg

			dairy or fat content not specified) with and without energy restriction	(with and without energy restriction)	
**Booth 2015**	19	1246 with normal weight, overweight, obesity	3-12 mo Calcium intake by 482–2400 mg/d via dairy products (low fat, no fat dairy or fat content not specified) with energy restriction	Intervention vs. usual diet or moderate dairy or low–dairy or low–Ca diet (with energy restriction)	–0.32 (–0.93; 0.30) kg
**Stonehouse 2016**	17	864 with overweight/obesity (1 study MS; 1 study PCOS)	8-48 wk. Dairy foods (milk, mixed dairy or yogurt) and dairy supplements (average 51 g/d of whey fraction high in leucine or whey protein isolate) with energy restriction	Intervention vs. usual diet (differences in the amount of dairy in the intervention vs. control group: 2- 4 s/d)	–0.92 (–1.63; –0.20) kg
**Geng 2018**	19	2116 Not defined	1-36 mo 0.5-5.24 g/d of dairy supplement without energy restriction	Intervention vs. usual diet or dairy–free diet or moderate serving dairy	0.36 (0.01; 0.70) kg
**Geng 2018**	16	930 Not defined	1-12 mo 0.5-5.24 g/d of dairy supplement with energy restriction	Intervention vs. usual diet or dairy–free diet or moderate serving dairy	–0.64 (–1.05; –0.24) kg
**Sochol 2019**	23	2362 with overweight, obesity	3-52 wk Dairy supplementation (especially low-fat dairies)	Intervention vs. control (i.e. usual diet; low (<2 s/d)	–0.42 (–0.72; –0.12) kg

				with or without an energy deficit or caloric restriction (dose NA)	or no dairy intake; placebo capsules; energy deficit diet; soy intake of a comparable energy intake)	
**LOW-FAT DAIRY**	**Benatar 2013**	8	941 with normal weight, overweight, obesity	≥ 1 mo Increased low fat dairy without additional interventions (dose NA)	Intervention vs. usual diet	0.82 (0.35; 1.28) kg
**FULL-FAT DAIRY**	**Benatar 2013**	10	688 with normal weight, overweight, obesity, MS	≥ 1 mo Increased whole fat dairy without additional interventions (dose NA)	Intervention vs. usual diet	0.41 (0.04; 0.79) kg
**Waist circumference**						
**DAIRY PRODUCT**	**Author, year**	**No. of studies**	**No. and type of subjects**	**Intervention (duration and dose)**	**Comparison**	Weighted Mean Difference (95 % CI)
**TOTAL DAIRY**	**Abargouei 2012**	2	147 Not defined	24 wk Increased dairy (3-5 s/d) without energy restriction	Intervention vs. usual diet	–2.68 (–8.02; 2.66) cm
	**Abargouei 2012**	7	289 Not defined	8-48 wk Increased dairy (3-5 s/d) with energy restriction	Intervention vs. usual diet	–2.43 (–3.42; –1.44) cm
	**Benatar 2013**	6	440 with normal weight, overweight, obesity, MS	4-16 wk (median 10 wk) Increased dairy without additional interventions (dose NA)	Intervention vs. usual diet	–0.07 (–1.24; 1.10) cm

	**Geng 2018**	5	555<br>Not defined	1-12 mo<br>0.5 - 5.24 g/d of dairy supplement without energy restriction	Intervention vs. usual diet or dairy–free diet or moderate serving dairy	–0.24 (–1.36; 0.89) cm
	**Geng 2018**	10	505<br>Not defined	2-12 mo<br>2.37-5.24 g/d of dairy supplement with and without energy restriction	Intervention vs. usual diet or dairy–free diet or moderate serving dairy	–2.18 (–4.30; –0.06) cm
	**Sochol 2019**	13	1348 with overweight, obesity	4-48 wk<br>Dairy supplementation (especially low-fat dairies) with or without an energy deficit or caloric restriction<br>(dose NA)	Intervention vs. control (i.e. usual diet; low (<2s/d) or no dairy intake; placebo capsules; energy deficit diet; soy intake of a comparable energy intake	–1.09 (–1.68; –0.58) cm
**LOW-FAT DAIRY**	**Benatar 2013**	2	134 with normal weight, overweight, obesity	≥ 1 mo<br>Increased low fat dairy without additional interventions (dose NA)	Intervention vs. usual diet	1.18 (–0.61; 2.96) cm
**FULL-FAT DAIRY**	**Benatar 2013**	4	306 with normal weight, overweight, obesity, MS	≥ 1 mo<br>Increased whole fat dairy without additional interventions (dose NA)	Intervention vs. usual diet	–0.58 (–1.99; 0.83) cm
**FERMENTED DAIRY OR DAIRY PLUS PROBIOTICS**	**Companys 2020**	6	511 with overweight, obesity	Dairy matrix added with probiotics (duration and dose NA)	Intervention vs. placebo or fermented milk	–0.49 (–0.68; –0.29) cm

CI: confidence interval; MS: metabolic syndrome; PCOS: polycystic ovarian syndrome; BMI: body mass index; wk: week(s); mo: month(s); s/d: serving per day; NA: not available.

Table S2. Summary of meta-analyses of randomized controlled trials on the effect of dairy products intake on fasting glucose, glycated hemoglobin and insulin resistance.

Fasting glucose						
**DAIRY PRODUCT**	**Author, year**	**No. of studies**	**No. and type of subjects**	**Intervention (duration and dose)**	**Comparison**	**Weighted Mean Difference (95% CI)**
**TOTAL DAIRY**	**Benatar 2013**	8	750 with normal weight, overweight, obesity, MS	8-12 wk (median 10 wk) Increased dairy without additional interventions (dose NA)	Intervention vs. usual diet	1.32 (0.19; 2.45) mg/dl
	**O' Connor 2019**	34	2678 with normal weight, overweight, obesity, MS, sarcopenia, hypertension	2-48 wk Elevated dairy intake (3.1 s/d)	Intervention vs. minimal intake (0.5 s/d)	0.07 (0.01; 0.12) mmol/L
**LOW-FAT DAIRY**	**Benatar 2013**	4	398 with normal weight, overweight, obesity	≥ 1 mo Increased low fat dairy without additional interventions (dose NA)	Intervention vs. usual diet	0.83 (–0.78; 2.43) mg/dL
	**O'Connor 2019**	14	862 Not defined	Elevated low fat dairy intake (< 2% fat content) (duration and dose NA)	Intervention vs. minimal intake	0.07 (0.00; 0.14) mmol/L
**FULL-FAT DAIRY**	**Benatar 2013**	4	352 with normal weight, overweight, obesity, MS	≥ 1 mo Increased whole fat dairy without additional interventions (dose NA)	Intervention vs. usual diet	1.80 (0.21; 3.40) mg/dL
**CHEESE**	**O'Connor 2019**	3	231 Not defined	Elevated cheese intake (duration and dose NA)	Intervention vs. minimal intake	–0.04 (–0.23; 0.14) mmol/L

**MILK AND/OR YOGURT**	**O’Connor 2019**	16	1136<br>Not defined	Elevated milk/yogurt intake<br>(duration and dose NA)	Intervention vs. minimal intake	0.08 (0.02; 0.14) mmol/L
**FERMENTED DAIRIES OR DAIRIES PLUS PROBIOTICS**	**O’Connor 2019**	8	535<br>Not defined	Elevated fermented dairy products intake (yogurt, cheese)	Intervention vs. minimal intake	0.02 (–0.09; 0.12) mmol/L
	**Companys 2020**	6	378 with type 2 diabetes	Dairy matrix added with probiotics (duration and dose NA)	Intervention vs. placebo or fermented milk	–0.37 (–0.58; –0.17) mmol/L
	**Dixon 2020**	5	304 with overweight, obesity, pre-type 2 diabetes, type 2 diabetes, MS, hypertension, CHD, hypercholesterolemia	Yogurt added with probiotics (duration and dose NA)	Intervention vs. no treatment or placebo product	–12.88 (–24.54; –1.22) mg/dl
	**Dixon 2020**	4	233 with overweight, obesity, pre-type 2 diabetes, type 2 diabetes, MS, hypertension, CHD, hypercholesterolemia	Milk added with probiotics (duration and dose NA)	Intervention vs. no treatment or placebo product	–7.54 (–23.84; 8.76) mg/dL
			**Glycated hemoglobin**			
**DAIRY PRODUCT**	**Author, year**	**No. of studies**	**No. and type of subjects**	**Intervention (duration and dose)**	**Comparison**	Weighted Mean Difference<br>(95% CI)
**TOTAL DAIRY**	**O’ Connor 2019**	4	512 with normal weight, overweight, obesity, MS	4-24 wk<br>Elevated dairy intake<br>(2.7 s/d)	Intervention vs. minimal intake (0.8 s/d)	–0.09 (–0.16; –0.03) %
	**O’ Connor 2019**	2	274 with MS	≥ 24 wk<br>Elevated dairy intake<br>(dose NA)	Intervention vs. minimal intake	–0.10 (–0.18; –0.02) %
**LOW-FAT DAIRY**	**O’Connor 2019**	2	238<br>Not defined	< 24 wk<br>Elevated low fat dairy intake (< 2% fat content)	Intervention vs. minimal intake	–0.08 (–0.20; 0.03) %

				(dose NA)		
**MILK AND/OR YOGURT**	**O'Connor 2019**	3	438<br>Not defined	Elevated milk/yogurt intake<br>(duration and dose NA)	Intervention vs. minimal intake	–0.09 (–0.16; –0.03) %
**FERMENTED DAIRIES OR DAIRIES PLUS PROBIOTICS**	**Dixon 2020**	5	325 with overweight, obesity, pre-type 2 diabetes, type 2 diabetes, MS, hypertension, CHD, hypercholesterolemia	Yogurt added with probiotics (duration and dose NA)	Intervention vs. no treatment or placebo product	–0.55 (–1.03; –0.06) %
	**Dixon 2020**	2	158 with overweight, obesity, pre-type 2 diabetes, type 2 diabetes, MS, hypertension, CHD, hypercholesterolemia	Milk added with probiotics (duration and dose NA)	Intervention vs. no treatment or placebo product	–0.61 (–0.81; 0.58) %
				**Insulin resistance (HOMA-IR)**		
**DAIRY PRODUCT**	**Author, year**	**No. of studies**	**No. and type of subjects**	**Intervention (duration and dose)**	**Comparison**	Weighted Mean Difference<br>(95% CI)
**TOTAL DAIRY**	**Benatar 2013**	4	270 with normal weight, overweight, obesity, MS	4-16 wk (median 10 wk)<br>Increased dairy without additional interventions (dose NA)	Intervention vs. usual diet	–0.63 (–1.50; 0.24)
	**O' Connor 2019**	13	840 with normal weight, overweight, obesity, MS, sarcopenia	4-24 wk<br>Elevated dairy intake (2.7 s/d)	Intervention vs. minimal intake (0.3 s/d)	–0.07 (–0.26; 0.12)
	**Sochol 2019**	12	794 with overweight, obesity	4-48 wk<br>Dairy supplementation (especially low-fat dairies) with or without an energy deficit or caloric restriction (dose NA)	Intervention vs. control (i.e. usual diet; low (<2s/d) or no dairy intake; placebo capsules; energy deficit diet; soy intake of a comparable energy intake)	–1.21 (–1.74; –0.67)

**LOW-FAT DAIRY**	**O'Connor 2019**	4	212<br>Not defined	≥ 12 wk<br>Elevated low fat dairy intake<br>(< 2% fat content)<br>dose NA)	Intervention vs. minimal intake	0.01 (–0.26; 0.28)
**CHEESE**	**O'Connor 2019**	2	131<br>Not defined	Elevated cheese intake<br>(duration and dose NA)	Intervention vs. minimal intake	0.24 (–0.19; 0.66)
**MILK AND/OR YOGURT**	**O'Connor 2019**	6	269<br>Not defined	Elevated milk/yogurt intake<br>(duration and dose NA)	Intervention vs. minimal intake	–0.01 (–0.26; 0.23)
**FERMENTED DAIRIES OR DAIRIES PLUS PROBIOTICS**	**O'Connor 2019**	4	188<br>Not defined	Elevated fermented dairy products intake<br>(yogurt, cheese)<br>(duration and dose NA)	Intervention vs. minimal intake	0.10 (–0.23; 0.43)

CI: confidence interval; CHD: coronary heart disease; MS: metabolic syndrome; HOMA-IR: homeostasis model assessment-insulin resistance; wk: week(s); mo: month(s); s/d: serving per day; NA: not available.

Table S3. Summary of meta-analyses of randomized controlled trials on the effect of dairy products intake on blood pressure.

			**Systolic blood pressure**			
**DAIRY PRODUCT**	**Author, year**	**No. of studies**	**No. and type of subjects**	**Intervention (duration and dose)**	**Comparison**	**Weighted Mean Difference (95% CI)**
**TOTAL DAIRY**	**Benatar 2013**	7	711 with normal weight, overweight, obesity, MS	7-26 wk Increased dairy without additional interventions (dose NA)	Intervention vs. usual diet	–0.41 (–1.63; 0.81) mmHg
**LOW-FAT DAIRY**	**Benatar 2013**	3	342 with normal weight, overweight, obesity, MS	≥ 1 mo Increased low fat dairy without additional interventions (dose NA)	Intervention vs. usual diet	–0.85 (–2.55; 0.84) mmHg
**FULL-FAT DAIRY**	**Benatar 2013**	4	369 with normal weight, overweight, obesity, MS	≥ 1 mo Increased whole fat dairy without additional interventions (dose NA)	Intervention vs. usual diet	0.07 (–1.69; 1.83) mmHg
**FERMENTED DAIRY OR DAIRY PLUS PROBIOTICS**	**Usinger 2012**	15	1232 with normal or high normal blood pressure or hypertension	4-21 wk Fermented milk	Intervention vs. placebo	–2.45 (–4.30; –0.60) mmHg
	**Dong 2013**	6	No. NA mostly with no antihypertensive drug use	8 wk (median) 100-450 g/d of probiotic fermented milk	Intervention vs. placebo product	–2.09 (–4.34; 0.17) mmHg
	**Dixon 2020**	3	217 with overweight, obesity, pre-type 2 diabetes, type 2 diabetes, MS, hypertension, CHD, hypercholesterolemia	Yogurt added with probiotics (duration and dose NA)	Intervention vs. no treatment or placebo product	–3.56 (–7.43; 0.31) mmHg
	**Dixon 2020**	4	214 with overweight, obesity, pre-type 2 diabetes, type 2 diabe-	Milk added with probiotics (duration and dose NA)	Intervention vs. no treatment or placebo product	–2.68 (–5.81; 0.44) mmHg

			tes, MS, hypertension, CHD, hypercholesterolemia			
**Diastolic blood pressure**						
**DAIRY PRODUCT**	**Author, year**	**No. of studies**	**No. and type of subjects**	**Intervention (duration and dose)**	**Comparison**	**Weighted Mean Difference (95% CI)**
**TOTAL DAIRY**	**Benatar 2013**	7	711 with normal weight, overweight, obesity, MS	7-26 wk Increased dairy without additional interventions (dose NA)	Intervention vs. usual diet	–0.45 (–1.70; 0.80) mmHg
**LOW-FAT DAIRY**	**Benatar 2013**	3	342 with normal weight, overweight, obesity, MS	≥ 1 mo Increased low fat dairy without additional interventions (dose NA)	Intervention vs. usual diet	–0.43 (–1.68; 0.82) mmHg
**FULL-FAT DAIRY**	**Benatar 2013**	4	369 with normal weight, overweight, obesity, MS	≥ 1 mo Increased whole fat dairy without additional interventions (dose NA)	Intervention vs. usual diet	–0.69 (–3.20; 1.83) mmHg
**FERMENTED DAIRY OR DAIRY PLUS PROBIOTICS**	**Usinger 2012**	15	1232 with normal or high normal blood pressure or hypertension	4-21 wk Fermented milk	Intervention vs. placebo	–0.67 (–1.48; 0.14) mmHg
	**Dong 2013**	5	No. NA mostly with no antihypertensive drug use	8 wk (median) 100-450 g/d of probiotic fermented milk	Intervention vs. placebo	–1.45 (–4.04; 1.14) mmHg
	**Dixon 2020**	3	217 with overweight, obesity, pre-type 2 diabetes, type 2 diabetes, MS, hypertension, CHD, hypercholesterolemia	Yogurt added with probiotics (duration and dose NA)	Intervention vs. no treatment or placebo	–3.54 (–5.13; –1.96) mmHg
	**Dixon 2020**	4	214 with overweight, obesity, pre-type 2 diabetes, type 2 diabetes, MS,	Milk added with probiotics (duration and dose NA)	Intervention vs. no treatment or placebo	–1.97 (–4.52; 0.58) mmHg

			hypertension, CHD, hypercholesterolemia			

CI: confidence interval; CHD: coronary heart disease; MS: metabolic syndrome; wk: week(s); mo: month(s); g/d: grams per day; NA: not available.

Table S4. Summary of meta-analyses of randomized controlled trials on the effect of dairy products intake on plasma lipids.

**Total cholesterol**						
**DAIRY PRODUCT**	**Author, year**	**No. of studies**	**No. and type of subjects**	**Intervention (duration and dose)**	**Comparison**	**Weighted Mean Difference (95% CI)**
**TOTAL DAIRY**	**Schwingshackl 2018**	NA	NA	Dairy intake (duration and dose NA)	Intervention vs. fish intake	–0.01 (–0.51; 0.50) mmol/L
	**Schwingshackl 2018**	NA	NA	Dairy intake (duration and dose NA)	Intervention vs. red meat intake	0.02 (–0.46; 0.50) mmol/L
	**Schwingshackl 2018**	NA	NA	Dairy intake (duration and dose NA)	Intervention vs. SSBs intake	–0.03 (–0.44; 0.39) mmol/L
**CHEESE**	**de Goede 2015**	5	119 Not defined	2-6 wk Hard cheese intake (weighted mean difference cheese vs. butter intake: 145 g/d)	Intervention vs. butter	–0.28 (–0.36; –0.19) mmol/L
**FERMENTED DAIRY OR DAIRY PLUS PROBIOTICS**	**Sun 2015**	10	NA	4-16 wk probiotic milk or yogurt (dose NA)	Intervention vs. placebo	–0.31 (–0.42; –0.20) mmol/L
	**Cho 2015**	8	378 with normal or high total cholesterol	4-8 wk of probiotic milk containing live bacteria (dose NA)	Intervention vs. placebo	–6.8 (–11.7; –1.9) mg/dL
	**Cho 2015**	15	637 with high or normal total cholesterol and/or diabetes and/or pregnancy	3-9 wk of probiotic yogurt/cheese containing live bacteria (dose NA)	Intervention vs. placebo	–8.3 (–12.4; –4.1) mg/dL
	**Shimizu 2015**	NA	NA	Fermented milk products (duration and dose NA)	Intervention vs. placebo or no treatment	–0.22 (–0.33; –0.11) mmol/L

	**Companys 2020**	4	248 with hypercholesterolemia	Dairy matrix added with probiotics (duration and dose NA)	Intervention vs. placebo or fermented milk	–0.46 (–0.73; –0.19) mmol/L
	**Dixon 2020**	5	393 with overweight, obesity, pre-type 2 diabetes, type 2 diabetes, MS, hypertension, CHD, hypercholesterolemia	Yogurt added with probiotics (duration and dose NA)	Intervention vs. no treatment or placebo product	–9.48 (–16.17; –2.48) mg/dL
	**Dixon 2020**	9	398 with overweight, obesity, pre-type 2 diabetes, type 2 diabetes, MS, hypertension, CHD, hypercholesterolemia	Milk added with probiotics (duration and dose NA)	Intervention vs. no treatment or placebo product	–8.74 (–14.62; –2.86) mg/dL
	**Pourrajab 2020**	7	352 with mild to moderate hypercholesterolemia	4-7 wk of 300 g/d of probiotic yogurt	Intervention vs. ordinary yogurt	–8.73 (–15.98; –1.48) mg/dL
	**Ziaei 2021**	38	2237 with normal weight, overweight, obesity, MS, NASH, pre-type 2 diabetes, type 2 diabetes, hypercholesterolemia (mostly mild or borderline), pre-hypertension	2-24 wk<br>80 - 600 ml/d of probiotic fermented milk	Intervention vs. conventional yogurt or milk	–8.30 (–11.42; –5.18) mg/dL
				**LDL-cholesterol**		
**DAIRY PRODUCT**	**Author, year**	**No. of studies**	**No. and type of subjects**	**Intervention (duration and dose)**	**Comparison**	**Weighted Mean Difference (95% CI)**
**TOTAL DAIRY**	**Benatar 2013**	9	702 with normal weight, overweight, obesity, MS	4-26 wk (median 12 wk)<br>Increased dairy without additional interventions (dose NA)	Intervention vs. usual diet	1.85 (–2.89; 6.60) mg/dL
	**Schwingshackl 2018**	NA	NA	Dairy intake (duration and dose NA)	Intervention vs. fish intake	–0.01 (–0.45; 0.42) mmol/L

	**Schwingshackl 2018**	NA	NA	Dairy intake (duration and dose NA)	Intervention vs. red meat intake	–0.02 (–0.43; 0.39) mmol/L
	**Schwingshackl 2018**	NA	NA	Dairy intake (duration and dose NA)	Intervention vs. SSBs intake	–0.03 (–0.37; 0.31) mmol/L
**LOW-FAT DAIRY**	**Benatar 2013**	3	342 with normal weight, overweight, obesity	≥ 1 mo Increased low fat dairy without additional interventions (dose NA)	Intervention vs. usual diet	–1.42 (–4.74; 1.91) mg/dL
**FULL-FAT DAIRY**	**Benatar 2013**	6	360 with normal weight, overweight, obesity, MS	≥ 1 mo Increased whole fat dairy without additional interventions (dose NA)	Intervention vs. usual diet	3.30 (–4.30; 10.90) mg/dL
**CHEESE**	**de Goede 2015**	5	119 Not defined	2-6 wk Hard cheese intake (weighted mean difference cheese vs. butter intake: 145 g/d)	Intervention vs. butter	–0.22 (–0.29; –0.14) mmol/L
**FERMENTED DAIRY OR DAIRY PLUS PROBIOTICS**	**Sun 2015**	11	NA	4-16 wk of probiotic milk or yogurt (dose NA)	Intervention vs. placebo	–0.47 (–0.70; –0.25) mmol/L
	**Cho 2015**	8	378 with normal or high total cholesterol	4-8 wk of probiotic milk containing live bacteria (dose NA)	Intervention vs. placebo	–9.2 (–13.4; –4.9) mg/dL
	**Cho 2015**	15	637 with high or normal total cholesterol and/or diabetes and/or pregnancy	3-9 wk of probiotic yogurt/cheese containing live bacteria (dose NA)	Intervention vs. placebo	–7.6 (–11.5; –3.6) mg/dL
	**Shimizu 2015**	10	397 with normal to moderate hypercholesterolemia	Fermented milk products (duration and dose NA)	Intervention vs. placebo or no treatment	–0.23 (–0.34; –0.13) mmol/L
	**Companys 2020**	4	248 with hypercholesterolemia	Dairy matrix added with probiotics (duration and dose NA)	Intervention vs. placebo or fermented milk	–0.50 (–0.77; –0.22) mmol/L
	**Dixon 2020**	4	370 with overweight, obesity, pre-type 2 diabetes, type	Yogurt added with probiotics (duration and dose NA)	Intervention vs. no treatment or placebo	–18.06 (–24.88; –11.24) mg/dL

			2 diabetes, MS, hypertension, CHD, hypercholesterolemia			
	**Dixon 2020**	6	287 with overweight, obesity, pre-type 2 diabetes, type 2 diabetes, MS, hypertension, CHD, hypercholesterolemia	Milk added with probiotics (duration and dose NA)	Intervention vs. no treatment or placebo	–9.82 (–18.28; –1.36) mg/dL
	**Pourrajab 2020**	7	352 with mild to moderate hypercholesterolemia	4-7 wk of 300 g/d of probiotic yogurt	Intervention vs. ordinary yogurt	–10.61 (–16.52; –4.69) mg/dL
	**Ziaei 2021**	36	1065 with normal weight, overweight, obesity, MS, NASH, pre-type 2 diabetes, type 2 diabetes, hypercholesterolemia (mostly mild or borderline), pre-hypertension	2-24 wk<br>80 to 600 ml/d of probiotic fermented milk	Intervention vs. conventional yogurt or milk	–7.34 (–10.04; –4.65) mg/dL
			**HDL-cholesterol**			
**DAIRY PRODUCT**	**Author, year**	**No. of studies**	**No. and type of subjects**	**Intervention (duration and dose)**	**Comparison**	**Weighted Mean Difference (95 % CI)**
**TOTAL DAIRY**	**Benatar 2013**	8	664 with normal weight, overweight, obesity, MS	4-26 wk (median 12 wk)<br>Increased dairy without additional interventions<br>(dose NA)	Intervention vs. usual diet	–0.19 (–2.10; 1.71) mg/dL
**LOW-FAT DAIRY**	**Benatar 2013**	3	342 with normal weight, overweight, obesity	≥ 1 mo<br>Increased low fat dairy without additional interventions<br>(dose NA)	Intervention vs. usual diet	0.73 (–2.50; 3.96) mg/dL

**FULL-FAT DAIRY**	**Benatar 2013**	6	360 with normal weight, overweight, obesity, MS	≥ 1 mo<br>Increased whole fat dairy without additional interventions (dose NA)	Intervention vs. usual diet	–0.69 (–3.04; 1.67) mg/dL
**CHEESE**	**de Goede 2015**	5	119<br>Not defined	2-6 wk<br>Hard cheese intake (weighted mean difference cheese vs. butter intake: 145 g/d)	Intervention vs. butter	–0.05 (–0.09; –0.02) mmol/L
**FERMENTED DAIRY OR DAIRY PLUS PROBIOTICS**	**Sun 2015**	7	NA	4-16 wk of probiotic milk or yogurt (dose NA)	Intervention vs. placebo	0.02 (–0.01;0.05) mmol/L
	**Cho 2015**	8	378 with normal or high total cholesterol	4-8 wk of probiotic milk containing live bacteria (dose NA)	Intervention vs. placebo	–2.1 (–4.5; 0.3) mg/ dL
	**Cho 2015**	15	637 with high or normal total cholesterol and/or diabetes and/or pregnancy	3-9 wk of probiotic yogurt/cheese containing live bacteria (dose NA)	Intervention vs. placebo	0.8 (–0.6; 2.1) mg/ dL
	**Companys 2020**	4	248 with hypercholesterolemia	Dairy matrix added with probiotics (duration and dose NA)	Intervention vs. placebo or fermented milk	0.26 (0.01; 0.52) mmol/L
	**Dixon 2020**	4	370 with overweight, obesity, pre-type 2 diabetes, type 2 diabetes, MS, hypertension, CHD, hypercholesterolemia	Yogurt added with probiotics (duration and dose NA)	Intervention vs. no treatment or placebo	0.25 (–1.82; 2.32) mg/ dL
	**Dixon 2020**	8	363 with overweight, obesity, pre-type 2 diabetes, type 2 diabetes, MS, hypertension, CHD, hypercholesterolemia	Milk added with probiotics (duration and dose NA)	Intervention vs. no treatment or placebo	0.86 (–2.07; 3.80) mg/ dL

	**Pourrajab 2020**	7	352 with mild to moderate hypercolesterolemia	4-7 wk of 300 g/d of probiotic yogurt	Intervention vs. ordinary yogurt	1.56 (–1.58; 4.71) mg/ dL
	**Ziaei 2021**	38	2267 with normal weight, overweight, obesity, MS, NASH, pre-type 2 diabetes, type 2 diabetes, hypercholesterolemia (mostly mild or borderline), pre-hypertension	2-24 wk 80-600 ml/d of probiotic fermented milk	Intervention vs. conventional yogurt or milk	0.07 (–0.73; 0.87) mg/ dL
			**Triglycerides**			
**DAIRY PRODUCT**	**Author, year**	**No. of studies**	**No. and type of subjects**	**Intervention (duration and dose)**	**Comparison**	**Weighted Mean Difference (95% CI)**
**TOTAL DAIRY**	**Schwingshackl 2018**	NA	NA	Dairy intake (duration and dose NA)	Intervention vs. fish intake	0.17 (–0.10; 0.45) mmol/L
	**Schwingshackl 2018**	NA	NA	Dairy intake (duration and dose NA)	Intervention vs. red meat intake	0.09 (–0.18; 0.36) mmol/L
	**Schwingshackl 2018**	NA	NA	Dairy intake (duration and dose NA)	Intervention vs. SSBs intake	–0.13 (–0.42; 0.16) mmol/L
**CHEESE**	**de Goede 2015**	5	119 Not defined	2-6 wk Hard cheese intake (weighted mean difference cheese vs. butter intake: 145 g/d)	Intervention vs. butter	–0.008 (–0.064; 0.049) mmol/L
**FERMENTED DAIRY OR DAIRY PLUS PROBIOTICS**	**Sun 2015**	9	NA	4-16 wk of probiotic milk or yogurt (dose NA)	Intervention vs. placebo	–0.02 (–0.18; 0.33) mmol/L
	**Cho 2015**	8	378 with normal or high total cholesterol	4-8 wk of probiotic milk containing live bacteria (dose NA)	Intervention vs. placebo	3.8 (–2.0; 0.95) mg/ dL

**Cho 2015**	15	637 with high or normal total cholesterol and/or diabetes and/or pregnancy	3-9 wk of probiotic yogurt/cheese containing live bacteria (dose NA)	Intervention vs. placebo	–8.1 (–16.9; 0.74) mg/ dL
**Companys 2020**	3	172 with hypercholesterolemia	Dairy matrix added with probiotics (duration and dose NA)	Intervention vs. placebo or fermented milk	–0.46 (–0.75; –0.14) mmol/L
**Dixon 2020**	5	393 with overweight, obesity, pre-type 2 diabetes, type 2 diabetes, MS, hypertension, CHD, hypercholesterolemia	Yogurt added with probiotics (duration and dose NA)	Intervention vs. no treatment or placebo	–1.61 (–6.50; 9.71) mg/ dL
**Dixon 2020**	10	438 with overweight, obesity, pre-type 2 diabetes, type 2 diabetes, MS, hypertension, CHD, hypercholesterolemia	Milk added with probiotics (duration and dose NA)	Intervention vs. no treatment or placebo	–6.99 (–26.34; 12.36) mg/ dL

CI: confidence interval; NASH: nonalcoholic steatohepatitis; CHD: coronary heart disease; MS: metabolic syndrome; wk: week(s); mo: month(s); g: grams; d: day; NA: not available.

Table S5. Summary of meta-analyses of randomized controlled trials on the effect of dairy products intake on markers of subclinical inflammation.

DAIRY PRODUCT	Author, year	No. of studies	No. and type of subjects	Intervention (duration and dose)	Comparison	Weighted Mean Difference (95% CI)
**C-reactive protein**						
**TOTAL DAIRY**	**Benatar 2013**	6	451 with normal weight, overweight, obesity, MS	7-26 wk (median 19 wk) Increased dairy without additional interventions (dose NA)	Intervention vs. usual diet	–1.07 (–2.54; 0.39) mg/L
	**Moosavian 2020**	10	641 with normal weight, overweight, obesity, MS	Elevated dairy intake (duration and dose NA)	Intervention vs. low or no dairy intake	–0.24 (–0.35; –0.14) mg/L
**LOW-FAT DAIRY**	**Benatar 2013**	3	182 with overweight, obesity	≥ 1 mo Increased low dairy without additional interventions (dose NA)	Intervention vs. usual diet	–0.62 (–1.35; 0.11) mg/L
**FULL-FAT DAIRY**	**Benatar 2013**	3	269 with normal weight, overweight, obesity, MS	≥ 1 mo Increased whole fat dairy without additional interventions (dose NA)	Intervention vs. usual diet	–1.81 (–5.47; 1.84) mg/L
**MILK AND/OR YOGURT**	**Moosavian 2020**	5	NA	Elevated milk/yogurt intake (duration and dose NA)	Intervention vs. placebo	–0.95 (–1.65; 0.24) mg/L
**TNF- α**						
**TOTAL DAIRY**	**Moosavian 2020**	8	NA	Elevated dairy intake (duration and dose NA)	Intervention vs. low or no dairy intake	–0.66 (–1.23; –0.09) pg/mL
**MILK AND/OR YOGURT**	**Moosavian 2020**	3	NA	Elevated milk/yogurt intake (duration and dose NA)	Intervention vs. placebo	–0.37 (–0.56; -0.18) pg/mL
**IL-6**						
**TOTAL DAIRY**	**Moosavian 2020**	7	NA	Elevated dairy intake (duration and dose NA)	Intervention vs. low or no dairy intake	-0.74 (-1.36; - 0.12) pg/mL

**MILK AND/OR YOGURT**	**Moosavian 2020**	3	NA	Elevated milk/yogurt intake (duration and dose NA)	Intervention vs. placebo	–0.43 (–2.69; 1.83) pg/mL
			**Adiponectin**			
**TOTAL DAIRY**	**Moosavian 2020**	7	NA	Elevated dairy intake (duration and dose NA)	Intervention vs. low or no dairy intake	2.42 (0.17; 4.66) µg/mL
**MILK AND/OR YOGURT**	**Moosavian 2020**	3	NA	Elevated milk/yogurt intake (duration and dose NA)	Intervention vs. placebo	14.28 (5.81; 22.74) µg/mL

CI: confidence interval; MS: metabolic syndrome; TNF-α: tumor necrosis factor-α; IL-6: interleuchin-6; wk: week(s); mo: month(s); NA: not available.
